# Diseases of the Muscular or Fibrous System

**Published:** 1838-02-28

**Authors:** N. Chapman

**Affiliations:** Professor of the Theory and Practice of Physic, in the University of Pennsylvania


### MEDICAL EXAMINER.

DEVOTED TO MEDICINE, SURGERY AND THE COLLATERAL SCIENCES.

**EDITED by J. B. BIDDLE, M. D. and M. CLYMER, M. D.**

No. 5. PHILADELPHIA, WEDNESDAY, FEBRUARY 28, 1838. Vol. I.

### DISEASES OF THE MUSCULAR OR FIBROUS SYSTEM.

*By* N. Chapman, M. D., *Professor of the Theory and Practice of Physic, in the University of Pennsylvania.*

(Reported for the Medical Examiner.)

This system comprehends the muscles and their appendages or immediate connections, the tendons, fasciæ, aponeuroses, ligaments, and the sero-fibrous and muco-fibrous tissues.

The disease, with an account of which I shall commence, is

### ARTHRITIS, OR GOUT.

Both of these terms are very objectionable, and were it practicable, should be repudiated. As it plainly imparts, from its derivation, arthritis means simply an inflammation of a joint, and in this general sense, without discrimination, was used till modern times. To a disease so pervading, and peculiarly liable to fluctuation—occupying in turn, every part of the system, the exterior as well as the interior, where there is any muscular or fibrous tissue, and presenting the most striking modifications, it is, indeed, utterly inapplicable. The other appellation has been still more unhappily selected. Derived from the Latin *gutta*, a drop, or perhaps more directly from the French, *goutte*, it was adopted during the prevalence of the false pathology, which supposed the disease to be owing to some acrid humour dropped in the joint, creative of the irritation or phlogosis. To compensate, however, for the meanness and vulgarity of this bad epithet, we have had applied to the subdivisions of the disease, a set of titles full of sound and dignity, coming immediately out of the classical fountain of the Greek. Thus, when in the foot, it is called *podagra*,—in the hand, *chiragra*—in the elbow, *onagra* or *pechyagra*—in the knee, *genagra*—in the clavicles, *cliesagra*—in the humerus, *omagra*—in the spine, *rachisagra*—in the teeth, *dentagra*—in the tendons, *tenontagra*, &c. Excepting podagra, which has been rendered familiar, and consecrated by long and general usage, it were mere pedantry to retain any other of these jaw-breakers.

Nosologists usually divide the disease into tonic and atonic, or regular and irregular, with some distinctions hereafter to be noticed.

The paroxysm of regular acute gout sometimes comes on without any decisive warning. Twice I have seen it attack with the suddenness of an electric shock. In 1826, I was visiting a patient for supposed dyspepsia, who, in the act of putting on his coat, was seized with such poignant arthritic pain in the elbow, that he became excessively alarmed lest a dislocation had taken place.

Conversing sometime afterwards with a valetudinary lady, on her general health, she, without any previous admonition, screamed out from a similar affection of her foot.

We learn from Van Swieten, that he saw a robust man, so stricken with gout in descending from his coach, that he thought he had luxated his ankle, and Guilbert tells us, that, in the retreat of some French troops across a bridge, he witnessed an officer so violently assailed by the disease, that at once, all power of motion was lost. But usually it is announced by some preliminary indications of gastro-enteric disturbance.

Connected with a coated tongue, the appetite is diminished, or sometimes anormal, depraved, or craving, just before an attack; the stomach is vexed with acidities, flatulency, and other symptoms of indigestion, attended by a sense of weight and tension in the abdomen, costive, or occasionally disordered bowels, copious and pallid, though more generally very high coloured, and scanty urine, with, in some instances, an irritable bladder, or, as has been noticed, a blennorhœal discharge, and an urgency of the venereal propensity. There are also yawning and stretching of the limbs, drowsiness, much lassitude and fatigue, itching of the skin, and, in short, a very pervading, and in some manner indescribable derangement of diverse functions.

Nor does the mind escape this distempered condition. Generally its operations are dull, confused, and inefficient; sometimes so much so, that it cannot be applied to any purpose of study or business. Not less, on other occasions, is the temper affected, being irritable, sour, petulant, or irascible; and I have seen the spirits depressed into moping melancholy, productive of the darkest views of life. For many years, I attended a friend, one of the most wealthy and substantial merchants of this city, who was always thus affected previously to an attack, conceiting every sort of disaster in his fortunes and reputation, which illusion it was vain to endeavour to dispel—though instantly on the disease fixing itself on a joint, his natural gaiety returned, with increased hilarity, like that of incipient inebriation. Frequently in this fine phrensy, did he exclaim to me, on entering his room, "I feel inspired, and am only fitted to write poetry!"

As more immediately prelusive of a paroxysm, an unusual coldness of the feet and legs, a suppression of perspiration in them, numbness, or a sense of pricking along the whole of both of the lower extremities, or cramps of the muscles, are to be remarked.

Commonly indisposed in some of the modes noticed, the individual goes to bed, and, after a few hours of disturbed sleep, is awakened by the severity of the pain, at first fluctuating, though oftener in some joint, and particularly that of the great toe, or in the heel, instep, or in the whole foot; which, becoming at length more violent, is succeeded by throbbing or gnawing in the part. This becomes inflamed, swollen, and intensely florid, the veins being turgid, and such exquisite tenderness exists, that the slightest pressure, even that of the bed clothes, cannot be borne, and any agitation of, or an attempt to move the limb, is productive of the extremest agony.

The local affection is not independent of constitutional participation. Chills or rigors, anticipatory, simultaneously, or subsequently take place, leading to fever. The degree of reaction, however, is very different; being sometimes moderate, while in other cases, it is very considerable, marked by a hard, strong, voluminous pulse, hot, dry surface, loaded tongue, more or less cerebral disturbance, the temper singularly worried, fractious and impatient, with excessive jactitation and inquietude. Towards morning, however, he usually falls asleep, and a gentle vapoury perspiration breaking out, abates the paroxysm. Yet it may be otherwise, or it shall continue unremittingly for an indefinite period. Even when most mitigated, there is not entire relief. During the day, there is still a considerable harassment, and towards evening, an exacerbation. It is this succession of paroxysms which constitutes what is familiarly called a fit of the gout. These, however, gradually prove milder, till the disease goes off, and frequently by critical discharges from the skin, kidnies or bowels, while at the same time, the edematous inflammation of the joint subsides, the cuticle partially desquamates, and excepting some itching, rigidity, and lameness, there is restoration to a state of health, very often even better than that preceding the attack. Many are the occasions indeed, where a regular fit of the gout has operated to disperse a series of the most diversified afflictions, so that its sulutary tendency has become a very popular notion. That it may supplant other diseases is not improbable, though mostly, its beneficial effects are to be ascribed to the transposition of itself from the interior, where it had vexed and deranged various organs, to the exterior, fixing on some one or more joints, on which it wastes its force.

Entirely different is the result, however, when the metastasis is incomplete, or any degree of the original irritation within continues. The cure under such circumstances, necessarily partial, is followed by manifestations of lingering disorder of diverse structures, particularly of the alimentary canal, the liver, and nervous system; and hence, indigestion, constipation, bile and other vitiated secretions, vertigo, depression of spirits, with general wretchedness, or violent head-ache or wandering pains throughout the body, which condition sometimes suddenly terminates in a stroke of apoplexy or palsy.

The duration of a fit of the gout will be longer or shorter, according to circumstances. Being the first attack, it seldom exceeds a few days, and on each repetition, the continuance is lengthened, till ultimately weeks elapse before convalescence is established. It may happen too, that after the disease has subsided in one joint, it seizes on some other, perhaps the opposite one, and to run the same lingering course.

An attack at first recurs generally at some distant interval, perhaps once in two or three years. It then comes on annually, or semi-annually, spring and fall, at length more frequently, and is of longer duration, each succeeding fit, till in some instances, there is scarcely any exemption from it, except, perhaps, in the middle of summer.

Degenerating ultimately into the chronic state, to which in its progress, it is prone, it varies materially from the acute form, as well in the constitutional as the local affections. Much pravity of system is exhibited, and especially of the primæ viæ, and biliary organs, in the shape of obstinate dyspepsia, torpid bowels, or occasional diarrhœa, the stools denoting a want of the bile—deficient, turbid, or loaded urine, or gravelly deposits—dry, harsh, sallow or dingy skin, petulant, morose, irresolute temper, and sometimes, confirmed hypocondriasm. Nor do the contents of the thoraric cavity escape. There are often very enduring irritations of the mucous membrane of the lungs, from simple bronchitis to asthma, or the most oppressive dyspnœa: no inconsiderable cardiac disorders, and hydropic effusions may take place.

Connected with this impaired or ruined state of the constitution, there is usually less acute suffering in the paroxysm. Moderate during the day, it is exacerbated at night, though even then more of a harassing ache, than positive pain, and the part is pale or purplish, rather than red, and with greater edema. The disease is very apt to fluctuate, transitions rapidly taking place. No great fever prevails, and it is more irritative than inflammatory. It is common for the joints to lose their strength and flexibility, and become so stiff sometimes, as to be deprived of motion.

Little indurated swellings may arise in the articulations of the fingers, to which Haygarth has applied the title of nodosities. Concretions of a chalky appearance are likewise formed upon the joints, and calculous affections occur from a deposite of the same kind of matter, the uvate of soda, in the kidnies or bladder, which though fluid at first, become dry and firm at last as a stone.

Gout selects as subjects of attack, and chiefly out of the aristocracy, men of robust frame, of full and corpulent habits, and of phlogistic diathesis. The physical constitution predisposing to it has been more minutely described as consisting in a large head, very capacious chest, gross and heavy body, soft, solid, full, distended veins, thick skin, and big bones. Numerous are the exceptions, however, to this rule, and I have seen it in directly the reverse, or in the feeble and attenuated; very frequently in women, and once in a boy of thirteen years of age. The latter is denied by Sydenham, Heberden, and the writers very generally. On this point, the language of Hippocrates, which I take from the Latin version of his works, is very strong. "*Puer non laborat podagrâ, ante veneris usum.*"

It may be safely affirmed, that it is very seldom to be met with before the age of puberty, more generally commencing beyond the meridian of life, and in its regular forms, is chiefly restricted to the male sex. Eunuchs, according to Hippocrates, do not have it, which however is denied by Richter and some other modern authorities.

Gout is thought to be mainly an inheritance, the sins of the father being visited upon his children, to the third and fourth generations, and especially on those who neglect or despise the precepts of sobriety and temperance. It is, to use the language of a late writer, as regularly transmitted in this way from the parent to his progeny, in many instances, as any species of property. But it may also be acquired. Thus as Shakspeare has said of honours, some acquire gout, and some have gout thrust upon them. Come however as it may, even "with the boast of heraldry or pomp of power," it is a most unwelcome guest.

Nevertheless, the estimate of its hereditary nature seems not to be founded, to the extent commonly received. To this point, a diligent inquiry has been directed by Scudamore; the result of which is, that the hereditary exceed only about one-third the acquired cases.

As so large a portion of attacks may be generated, it is right that the causes producing the disease, should be pointed out with some particularity, and the more so, since by the avoidance of them, the origination, as well as the development of the disease, when hereditary, may at least, in some instances, be prevented.

Of these, by far the most prolific is an excess in eating and drinking, aided by sedentary habits. No drink seems to be so pernicious in this respect as wine. It is said, that while ardent spirits derange more directly the hepatic apparatus in various ways, wine has the effect of exciting the arthritic affections. But some wines do this more than others. An opinion is very generally entertained in Great Britain, and in which we mostly concur in this country, that claret and other light wines, are particularly pernicious. But the French, the Spaniards, the Italians, and the people of the north of Europe, who chiefly use such wines, are comparatively exempt from the disease; while in Britain, where they are much less consumed, it prevails, perhaps, more than in any section of the world.

That Port, the principal wine of the people of that country, is the most unwholesome of all wines, even in a state of purity, I have no doubt whatever, as well from its peculiar qualities, as from actual experience of its effects in those who use it freely. But factitious and adulterated as nearly the whole of it is confessedly in Britain, it must *a fortiori* prove exceedingly mischievous, and is probably one of the chief causes of the wide-spread prevalence there of arthritic affections.

The same contradictory statements prevail as to malt liquors. By some, as Sydenham, Van Swieten, Linnæus, &c., they are supposed to be rather preventive in their effects. Yet the preponderance of authority is on the other side; and certain it is, that the disease has augmented in England, since the common consumption of these beverages.

Of cider, the denunciation of its use is nearly universal in relation to gout. But here too, there are some opposing facts. Cider is the common drink of the people of New England, among whom the disease is of rare occurrence;—whereas, in the Southern states, it is far more general, though in place of this beverage, ardent spirits and Madeira wine are usually consumed.

More pernicious than any I have mentioned, is perhaps the habitual use of punch or lemonade, or any other drink prepared from citric acid. It may, perhaps, be safest to infer on the whole, that the abuse of any of these articles is, by disordering the digestive organs, productive in a greater or less degree of this disease.

Not less operative, in the same way, is an improper indulgence in eating. It is held by some of our highest authorities to be even more so, in the case of high-seasoned and stimulating food habitually taken, and which is sufficiently probable, when we advert to its ultimately debilitating and deranging effects on the stomach and its connections.

Of the dependence of gout on the habits of living, no stronger proof can probably be supplied, than from the annals of this city. Not longer ago than when I commenced my professional career, the disease abounded in the higher circles. Then it was the practice to drink punch in the forenoon, to continue it at dinner, or to resort to ardent or malt liquors, followed by a liberal use of diverse wines, closing the evening with substantial suppers and stimulating potations. But in this respect, within the last fifteen or twenty years, a signal change has taken place. No punch or distilled spirits, and comparatively little malt liquor has been consumed, and the custom of supping is nearly extinct. Temperance has superceded debauchery and excess, and gout thus deprived of its aliment, is fast perishing away. It is a fact, which my ample opportunities have enabled me to ascertain, that so late as the commencement of the present century, a hundred cases of the disease existed in this community, where one is now to be met with, and with few exceptions, these are the remnants of other days, serving as monuments of a state of society, of which there are scarcely any other traces to be recognised.

To the causes of gout must be added sedentary, indolent, or intensely studious habits.

It is well known, that in the common orders of life, the disease hardly exists, and infinitely less among the higher classes who pursue occupations of active industry. Neglect of the exercise of walking has particularly an effect. We are told, that the inhabitants of Edinburgh, who, from their wealth, or the location of their trade at Leith, the sea-port town, a mile or more distant, ride usually, are very liable to the disease;—while those of Glasgow, the practice of whom is different, are nearly exempt from it.

Men of great intellectual application and distinction, have been singularly prone to it, of which examples might be cited from the history of all ages.

Treating of this disease, Sydenham, who was a martyr to it, says—"But what is a consolation to me, and may be so to other *gouty* persons of small fortune and slender abilities, is, that kings, princes, generals, admirals, philosophers, and several other great men, have thus lived and died: in short, it may in a more especial manner be affirmed of this disease, that it destroys more rich than poor, more wise men than fools, and, which seems to demonstrate the justice and strict impartiality of Providence, who abundantly supplies those that want some of the conveniences of life, with other advantages, and tempers its profusion to others, with equal mixture of good and evil, so adapted to our weakness and perishable condition, as is perhaps admirably suited to the present state."

As concerned in the predisposition, it would seem that climate has some claims to be mentioned. The disease is rarer in the tropical and hyperborean, than the temperate regions, and especially where the latter are humid and austere. Much depends, undoubtedly, on the habits of the people, though,cœteris paribus, there is said to be a material difference. Certain it is, that in this city at least, the seasons of the most frequent occurrence of the disease, are early in the spring and late in the fall, when the weather is damp and chilly.

An hereditary or acquired tendency existing, the paroxysm may be excited by numerous circumstances, such as grief, vexation or any anxiety of mind, the indulgence of violent passions, particularly the rage of anger, which indeed is quaintly called by an old writer, "the midwife of gout," from its so frequently bringing it forth. To this may be added, inordinate venery, much fatigue, and the loss of sleep, exposure to cold, especially the feet, or compression of these by tight boots or shoes—or strains or contusion of a joint, the sudden change from a full to an extremely low diet, and above all, by particular articles of food or drink. The acescent, flatulent vegetables or fruits will sometimes induce an attack very promptly, and still more so, the sour beverages. Lemonade I have heard to do it in a few minutes; in one of my late friends, Mr. Francis, a simple glass of claret or champaigne had the same effect; and another, the late General Robinson, informed me, that even the odour of cider, had often brought it on in him.

(*To be continued.*)

